# A comprehensive diagnostic service to clarify intervention needs when work participation is at risk: study protocol of a randomized controlled trial (GIBI, DRKS00027577)

**DOI:** 10.1186/s12913-022-08513-1

**Published:** 2022-09-09

**Authors:** David Fauser, Saskia Dötsch, Claudia Langer, Vera Kleineke, Claudia Kindel, Matthias Bethge

**Affiliations:** 1 Institute for Social Medicine and Epidemiology, University of Lübeck, Ratzeburger Allee 160, 23562 Lübeck, Germany; 2 Fachklinik Aukrug, Tönsheide 10, 24613 Aukrug, Germany; 3 RehaCentrum Hamburg, Heidenkampsweg 41, 20097 Hamburg, Germany; 4 German Pension Insurance North, Ziegelstraße 150, 23556 Lübeck, Germany; 5 Rostocker Zentrum für ambulante Rehabilitation, Wismarsche Str. 32, 18057 Rostock, Germany

**Keywords:** Employment, Diagnostic service, Work ability, Occupational health physicians, Patient reported outcome measures, Randomized controlled trial

## Abstract

**Background:**

Effective care services for people whose work participation is at risk require low-threshold access, a comprehensive diagnostic clarification of intervention needs, a connection to the workplace and job demands, and interdisciplinary collaboration between key stakeholders at the interface of rehabilitation and occupational medicine. We have developed a comprehensive diagnostic service to clarify intervention needs for employees with health restrictions and limited work ability: this service is initiated by occupational health physicians.

**Methods/design:**

Our randomized controlled trial tests the effectiveness of a comprehensive diagnostic service for clarifying intervention needs (GIBI: Comprehensive clarification of the need for intervention for people whose work participation is at risk). The comprehensive intervention comprises three elements: initial consultation, two-day diagnostics at a rehabilitation center and follow-up consultations. We will include 210 employees with health restrictions and limited work ability, who are identified by occupational health physicians. All individuals will receive an initial consultation with their occupational health physician to discuss their health, work ability and job demands. After this, half the individuals are randomly assigned to the intervention group and the other half to the waiting-list control group. Individuals in the intervention group start two-day diagnostics, carried out by a multi-professional rehabilitation team in a rehabilitation center, shortly after the initial consultation. The diagnostics will allow first recommendations for improving work participation. The implementation of these recommendations is supported by an occupational health physician in four follow-up consultations. The control group will receive the comprehensive two-day diagnostic service and subsequent follow-up consultations six months after the initial consultation. The primary outcome of the randomized controlled trial is self-rated work ability assessed using the Work Ability Score (0 to 10 points) six months after study inclusion. Secondary outcomes include a range of patient-reported outcomes regarding physical and mental health, impairment, and the physical and mental demands of jobs.

**Discussion:**

This randomized controlled trial is designed to test the effects of a new complex intervention involving a comprehensive clarification of intervention needs in order to promote work participation and prevent the worsening of health and work disability.

**Trial registration:**

German Clinical Trials Register (DRKS00027577, February 01, 2022).

**Supplementary Information:**

The online version contains supplementary material available at 10.1186/s12913-022-08513-1.

## Background

Employment is a key resource for participation in society [1–3]. It ensures income and material security, supports an independent lifestyle, and reduces the risk of poverty in old age by building up pension entitlements. Employment enables social contacts and experiences of self-efficacy, and it can give a sense of purpose and contribution [4]. If a job’s demands and a person’s work ability drift apart due to health problems (e.g., mental illness or high physical job demands), this can jeopardize future work participation and the ability to stay in the workplace. In order to sustainably improve the work ability and participation of people with health impairments, the Federal Ministry of Labour and Social Affairs of Germany initiated the grant program “Innovative Ways to Participate in Working Life – Rehapro”. The aim of Rehapro is to develop and improve rehabilitation services in order to prevent health-related early retirement. Our study focuses on the question: How can we reach people whose work participation is at risk through tailored interventions that are offered early enough to prevent chronification and work disability? We believe the following four factors are important in order to effectively support people, and that it is essential to consider them when developing an intervention to support work participation and prevent work disability.

First, there is a need for low-threshold access to care services and occupational health offers. Problems accessing rehabilitation services were identified in Germany. Around half of the persons granted a disability pension (i.e. benefits to reduce income losses in case of long-term and permanent work disability) have never used a medical rehabilitation service (i.e. a multidisciplinary program in order to maintain and restore work ability and to avoid disability pensions) [5, 6]. Participation in a medical rehabilitation program requires a claim by the person in need, and the personal support of primary care or occupational health physicians. A lack of knowledge about the range of supporting services offered by rehabilitation providers under German social law makes it difficult to find suitable interventions [7]. Social support from primary and occupational health physicians can be a contributing factor in applying for rehabilitation services [8]. A representative survey of 20,012 employed persons shows a discrepancy between the offer and use of workplace health promotions [9]. Interventions in workplace health promotion implemented in their company were reported by 47% of employees, however, only one in four employees had used an offer of workplace health promotion in the last two years [9]. Increasing the use of workplace interventions requires proactively addressing specific groups (e.g. individuals with known diagnoses, stressful factors at work or in private life, or with increasing periods of sick leave) [9].

Second, an individualized and comprehensive diagnostic clarification of health problems is necessary in order to be able to support affected persons. The initial focus when clarifying health needs for employees whose work participation is endangered is often on physical complaints, however, the actual problem is often complex, and has bio-psycho-social causes and consequences [10]. A comprehensive approach is necessary in order to understand the origin of risks to work participation, and to derive appropriate interventions. The bio-psycho-social model of the International Classification of Functioning, Disability and Health (ICF) provides a theoretical basis [11]. A review of meta-analysis and systematic reviews suggested that employees benefit particularly from multimodal workplace intervention strategies, including physical, psychological and social components [12].

Third, the effectiveness of interventions in improving work participation depends largely on whether they meet individual needs. Preventive and rehabilitative health measures are still strongly oriented towards symptoms of illness, without considering the requirements and stress factors of the workplace. An often-described criticism of medical rehabilitation is the lack of a connection to the workplace and its environment. This means that the recommendations made by rehabilitation centers cannot always be implemented in the workplace. A systematic review by van Vilsteren et al. [13] shows that the involvement of employers and the implementation of workplace adaptations in the reintegration process increase return to work rates and reduce sickness absence (14 randomized controlled trials, 1897 subjects). A systematic review also showed that early workplace-oriented interventions for individuals with short periods of sickness absence (10 to 84 days) were more effective and cost-effective than measures implemented later [14].

Fourth, occupational health physicians have an important linking function in the initiation and monitoring of rehabilitation processes due to their in-company knowledge and skills, and can support the return-to-work process directly in the workplace. However, the interface between occupational and rehabilitative care in Germany is characterized by insufficient communication and cooperation between rehabilitation and occupational health physicians [15]. Rehabilitation and occupational health physicians report organizational (e.g. poor availability), interpersonal (e.g. patient levels of trust in physicians) and structural (e.g. data protection regulations) barriers that make cooperation difficult and inadequate [16]. Multi-level stakeholder approaches are necessary to improve this cooperation. A previous project, GABI (Grundfos-Aukrug zur Erhaltung der Beruflichen Integration), successfully tested a multi-stakeholder collaboration between a company, occupational health physician and the multidisciplinary team of a rehabilitation center [10].

We evaluated this approach in our study in three model regions where we established networks between rehabilitation centers, companies and occupational health physicians. Our intervention comprises three modules: initial consultation with the occupational health physician, a two-day comprehensive diagnostic service with medical, physiotherapeutic and psychotherapeutic elements from a multi-professional team, and follow-up consultations with the occupational health physician. We expect that people with limited work ability will receive tailored support and services they need to improve their ability to work and their participation in working life, and to prevent chronification and work disability.

### Objectives

We designed a randomized controlled trial to determine whether a comprehensive diagnostic service for the clarification of intervention needs improved self-rated work ability six months after study inclusion compared to individuals in a control group starting the intervention six months later. The study also assesses how well the intervention is conducted by the occupational health physicians, and implemented in the three rehabilitation centers.

### Trial design

Our study is a randomized controlled trial with two parallel groups, comparing individuals in the intervention group with individuals in a waiting-list control group. Employees with health restrictions and limited work ability from the participating companies are randomly assigned to the intervention or waiting-list control group. The intervention consists of a comprehensive diagnostic service, initiated and followed-up by occupational health physicians. The waiting-list control group will receive the intervention six months after study inclusion. A similar approach was adopted in a randomized controlled trial analyzing the effectiveness of pulmonary rehabilitation in patients with asthma [17]. The use of a waiting-list control group allows all eligible individuals to participate in the intervention. We expect that this will support willingness to participate in our randomized controlled trial and eliminate potential reservations about randomized assignment among both participating companies and potential study participants.

Questionnaire data was used to analyze the effects of our intervention, and assessed at study inclusion (initial consultation) and six months later in both groups. We will also assess the therapy dose received and delivered at the end of the two-day comprehensive diagnostics. Individual interviews with participants supplement our study with qualitative data.

## Methods

### Study setting

Our intervention was implemented in three rehabilitation centers in the German federal states of Hamburg, Mecklenburg-Western Pomerania and Schleswig–Holstein in a pilot study. The three centers each have an orthopedic and a psychosomatic section. A network of occupational health physicians and companies is also involved in recruiting the study participants in our project. One study coordinator per rehabilitation center will coordinate the conduct of the study in the rehabilitation center, and manage the network with the companies and occupational health physicians.

### Eligibility criteria

We include employees with health restrictions and a limited ability to work, who have been on sick leave for at least four weeks in the past 12 months, have been employed in the cooperating companies for at least six months, and are insured with the German Pension Insurance North, Federal German Pension Insurance, German Pension Insurance Braunschweig-Hannover or German Pension Insurance Knappschaft-Bahn-See. Individuals about whom the occupational health physician was concerned and/or where there was other evidence that individual work ability and job demands were increasingly drifting apart, such as frequent periods of sick leave, will also be included.

We will exclude individuals who require urgent medical care due to acute illnesses, who have a clear need for rehabilitation services or who initially need support due to an addiction disorder. The occupational health physicians will inform these people about alternative services.

### Treatment

#### Intervention

The comprehensive intervention to clarify the need for intervention is initiated by occupational health physicians and comprises three elements: initial consultation, two-day comprehensive diagnostics, and follow-up consultations by the occupational health physician. There are no restrictions on concomitant care or interventions during the trial. Table 1 describes the three components of the comprehensive intervention strategy in line with the Template for Intervention Description and Replication (TIDieR) checklist [18].

**Table 1:** Description of the three components according to the TIDieR checklist

Brief name	Initial consultation	Two-day comprehensive diagnostics	Follow-up consultations
Why	The employee and the occupation health physician get to know each other. Any further questions about the intervention, study participation, data protection, confidentiality and the voluntary nature of participation are clarified during the initial consultation. The employee should make an informed decision about participating in the two-day diagnostics at the rehabilitation center. An initial needs analysis and joint goals for participation in the intervention should be developed based on the employee’s report of health and contextual factors. Finally, the occupational health physician needs to clarify the eligibility of the employee for study participation Occupational health physicians have an important linking function in the initiation and monitoring of rehabilitation processes due to their in-company knowledge and skills, and can support the return-to-work process directly in the workplace [19]	Diagnostic measures are needed to develop recommendations for adapting the employee’s job environment and to improve work participation. Comprehensiveness is necessary so as to understand the underlying health problem, which is often complex and has bio-psycho-social causes and consequences, and to derive appropriate interventions. Work-related assessments are carried out in order to plan and design therapeutic measures individually and to align them to the actual workplace Social counseling provides information on various possibilities for supporting work participation and individual occupational and social problems Coaching approaches in the final meeting and tests for optional therapeutic measures are used to improve the participant’s self-reflection skills, resilience, and motivation. A resource-based approach aims to activate available skills	The occupation health physician and the employee should discuss the recommendations for action derived from the rehabilitation center to secure employment and improve work ability. These recommendations are to be recorded during joint goal setting. The other stakeholders who need to be involved in realizing the recommendations need to be clarified Involving occupational health physicians in the rehabilitation and return-to-work process can improve the success of rehabilitation, increase sustainable stay-at-work and reduce sick leave [13]
What (materials)	Guidelines for initial one-on-one consultation; computerized documentation; questionnaire for the collection of medical data and job demands; “Analysis of needs and objectives” worksheet; study information; informed consent form (in duplicate); baseline questionnaire	Equipment at the rehabilitation center for the performance of work-related diagnostics; final report of the two-day diagnostics in the rehabilitation center; questionnaire at the end of the two-day diagnostics	Guidelines for follow-up consultations; “Recommendations and agreement on objectives” worksheet; computerized documentation; final report of the two-day diagnostics in the rehabilitation center
What (procedures)	The employee meets with the occupational health physician in the practice or their office, or directly in the company for an initial one-on-one consultation. The consultation includes the following elements: information and consent from the employee to participate in the study, completion of the baseline questionnaire, examination of the employee's current health limitations and work ability, and completion of a description of job demands. The completed documents are sent to the study coordinator in the rehabilitation center after the consultation	The program of the two-day diagnostics in the rehabilitation center is arranged by the study coordinator in consultation with the rehabilitation team on the basis of the patient information resulting from the initial consultation Physiotherapeutic and psychotherapeutic diagnostics, social counseling and a final meeting communicating the results to the participant are necessary elements of the two days. The physiotherapeutic diagnostics include an evaluation of functional capacity. The content is based on anamnestic symptoms and job demands. The psychotherapeutic diagnostics include psychosomatic exploration in order to identify pathological disorders, psychosomatic comorbidity and relevant functional limitations. The social counseling examines the individual’s work–life situation and provides socio-legal guidance and advice concerning further assistance within the social security system Other optional elements are provided in order to test therapeutic action possibilities and strengthen self-care competences. These include seminars on sports and exercise, nutrition, sleep hygiene, as well as psychoeducational groups on depression, pain or stress management, and participation in occupational therapy or autogenic training , Specific recommendations for action to maintain work participation are developed by the rehabilitation team in a case conference, and recorded in a final report	The occupational health physician and the employee have at least one and up to four follow-up consultations, as required, within five months after the two-day diagnostics. The occupation health physician accompanies the employee in realizing the recommendations for action that were developed based on the results of the two-day diagnostics. The first follow-up consultation should take place as soon as possible after the two-day diagnostics. The other three follow-up consultations are held at regular intervals to review and, if necessary, update, needs, goals and the involvement of other stakeholders. Questions from the occupational health physician can be clarified with the medical contact person at the rehabilitation center if necessary
Who (provided)	Occupational health physician	Interdisciplinary team at a rehabilitation center (i.e. physicians, physiotherapists, psychotherapists, occupational therapists and social counselors) and a study coordinator	Occupational health physician
How	In presence and individually	In presence and individually	In presence, digitally or by telephone and individually; if necessary, with the involvement of other stakeholders (e.g., employer or employee representative)
Where	In the practice of the occupational health physician or directly in the company	Inpatient or outpatient in one of three rehabilitation centers in the German states of Hamburg, Mecklenburg-Western Pomerania and Schleswig–Holstein	In the occupational health physician’s practice or directly in the company
When and how much	Once with a duration of up to two hours	Two days with an overall mean therapy dose of nine hours	Up to four consultations, with a duration of one hour each within five months of the end of the two-day diagnostics in the rehabilitation center
Tailoring	Not planned	The dose of the diagnostic measures is fixed. Individual treatments trials are tailored to the needs of the participants, which are derived from the initial consultation with the occupational health physician	As needed, up to four consultations can be conducted to implement the recommendations
How well	The initial one-on-one consultation is described in guidelines to ensure standardized implementation The initial one-on-one consultation is documented by the occupational health physician in a standardized manner. The occupational health physicians were trained to conduct the consultations and in the required computerized documentation before the randomized controlled trial began	All diagnostic components were developed by the interdisciplinary project team (i.e. rehabilitation center, research institution and German Pension Insurance North) in 2020 to ensure standardized implementation. The interdisciplinary team were trained to conduct a standardized comparison of job demands and individual work ability before the randomized controlled trial began Regular video conferences will be held with the study coordinators to support the accuracy of the intervention throughout the process. At the end of the two-day diagnostics and at the six-month follow-up, all participants are asked which intervention components they received during the intervention using standardized questionnaires	The follow-up consultations are described in guidelines to ensure standardized implementation The follow-up consultations are documented by the occupational health physician in a standardized manner. The occupational health physicians were trained to conducti the consultations, and in the required computerized documentation before the randomized controlled trial began
	Guided interviews will be conducted with participants during the follow-up consultation phase, asking which intervention elements participants received and how they could be improved, in order to assess treatment fidelity		

#### Control

The control group will receive the comprehensive two-day diagnostics and subsequent follow-up consultations with the occupational health physician six months after the initial occupational health consultation.

### Ancillary and post-trial care

There is no planned ancillary or post-trial care. There is also no plan for compensation for harms due to study participation.

### Outcomes and other measures

A complete list of all measured constructs, measurement points and the expected scaling of the randomized controlled trial can be found in Table 2. Adverse events will not be systematically assessed.

**Table 2:** Measures, assessment, expected scaling, and measurement time points in the randomized controlled trial

*Outcome*	*Source and reference*	*Scaling*	*Baseline*	*End of the two-day diagnostics*	*6-month follow-up*
**Primary outcome**					
Self-rated work ability	WAS [20, 21]	Continuous	X	X	X
**Secondary outcomes**					
General health	COPSOQ [22, 23]	Continuous	X	X	X
Depression	PHQ-4 [24, 25]	Continuous	X	X	X
Anxiety	PHQ-4 [24, 25]	Continuous	X	X	X
Physical functioning	RMQ [26, 27]	Continuous	X		X
Physical activity	BSA-F [28]	Continuous	X		X
Employment status	Own development	Binary	X		X
Sick leave	Own development	Binary	X		X
Sick leave duration in weeks	Own development	Continuous	X		X
Physical demands	FEBA [29]	Continuous	X		X
Mental job demands	COPSOQ [22, 23]	Continuous	X		X
Support by supervisor and colleagues	COPSOQ [22, 23]	Continuous	X		X
Working atmosphere	COPSOQ [22, 23]	Continuous	X		X
Job insecurity	COPSOQ [22, 23]	Continuous	X		X
Job satisfaction	COPSOQ [22, 23]	Continuous	X		X
Workplace bullying	COPSOQ [22, 23]	Continuous	X		X
**Other measures**					
Self-rated work ability	WAI [20, 21]	Continuous	X		
Self-evaluation of functional capacity	M-SFS [30, 31]	Continuous		X	
Outpatient visits to physicians	Own development	Continuous	X		
Use of outpatient therapy	Own development	Continuous			X
Use of rehabilitation	FIMA [32]	Binary			X
Job title	Own development	Nominal	X		X
Working hours	Own development	Ordinal	X		
Temporary work	Own development	Nominal	X		
Fixed-term job contracts	Own development	Nominal	X		
Shift work	Own development	Nominal	X		
Size of company	Own development	Nominal	X		
Sociodemographic data	Own development	Nominal/continuous	X		
Dose delivered: initial consultation	Computerized sheet (own development)	Binary/continuous		X	
Dose delivered: two-day diagnostics	Computerized sheet (own development)	Binary/continuous		X	
Dose delivered: follow-up consultations	Computerized sheet (own development)	Binary/continuous			X
Self-evaluation of functional capacity	Own development	Continuous		X	
Action skills	Own development	Continuous		X	
Subjective goal achievement	Own development	Continuous			X
Content of intervention	Own development	Continuous		X	X
Rating of intervention components	Own development	Continuous			X

*WAS* Work Ability Score, *COPSOQ* Copenhagen Psychosocial Questionnaire, *PHQ* Patient Health Questionnaire, *RMQ* Roland and Morris Disability Questionnaire, *BSA-F* Bewegungs- und Sportaktivität Fragebogen, *FEBA* Fragebogen zur subjektiven Einschätzung der Belastungen am Arbeitsplatz, *WAI* Work Ability Index, *M-SFS* Modified Spinal Function Sort, *FIMA* Fragebogen zur Inanspruchnahme medizinischer und nicht-medizinischer Versorgungsleistungen im Alter

#### Primary outcome

Our primary outcome is self-rated work ability using the single-item Work Ability Score (WAS), which is the first item of the Work Ability Index (WAI) and measures current compared with lifetime best work ability [20, 21]. The score ranges from 0 (completely unable to work) to 10 (work ability at its best). Higher scores indicate better self-rated work ability. The WAS is closely correlated with the entire Work Ability Index score [21], and predicts work disability and health-related early retirement [33–35]. The WAS is assessed during the initial consultation with the occupational health physician, and at the six-month follow-up, and also at the end of the two-day diagnostics.

#### Secondary outcomes

Our secondary outcomes with regard to health, physical functioning, mental health and employment are assessed in the initial consultation and at six-month follow-up, and also to some extent at the end of the two-day diagnostics.

##### General health

General health will be assessed with one item from the Copenhagen Psychosocial Questionnaire using an 11-point scale (0 ‘worst imaginable health state’ to 10 ‘best imaginable health state’) [22, 23]. General health is also assessed at the end of the two-day diagnostics.

##### Depression and anxiety

The two-item versions of both the depression module (PHQ-2) and the anxiety module (GAD-2) of the Patient Health Questionnaire (PHQ-4) will be used to assess depression and anxiety [24, 25]. All items are measured on a four-point scale (0 = not at all, 1 = several days, 2 = more than half of the days, 3 = nearly all days). Total scores for depression and anxiety range from zero to six points. We will also determine binary outcomes by categorizing values of > 2 as clinically relevant depressive or anxiety disorder. Information about depression and anxiety will also be collected at the end of the two-day diagnostics.

##### Physical functioning

Physical functioning is assessed using the Roland and Morris Disability Questionnaire (RMQ) [26, 27]. The RMQ consists of 24 items related to impairments with regard to activities of daily living. Participants are asked to state the items which describe their impairments. Each item is coded with 0 and 1, resulting in a total score of 0 to 24 points. A higher score indicates higher impairment and disability.

##### Physical activity 

Physical activity will be assessed using the German Physical Activity, Exercise, and Sport Questionnaire (Bewegungs- und Sportaktivität Fragebogen, BSA-F) [28]. We will assess the number of different exercise activities undertaken during the last four weeks and the frequency and duration in minutes of each activity. Frequency and duration are multiplied for each mention of activity. The products are summed to obtain a total physical activity index and divided by four to get the unit minutes per week.

##### Employment

We will assess employment state (employed vs. unemployed) to describe work participation. We will also ask for the number of weeks of sickness absence (current state and duration in the past six months).

##### Work stress and work environment 

Physical job demands will be measured using the questionnaire on job demands (Fragebogen zur subjektiven Einschätzung der Belastungen am Arbeitsplatz, FEBA) [29]. The FEBA consists of five 4-point items that yield a total score ranging from 0 to 15 points. Higher values indicating higher levels of job demands.

Psychological job demands (six items), job insecurity (two items), support by supervisor and colleagues (two items), atmosphere at work (one item), and overall job satisfaction (one item) will be assessed using the short version of the Copenhagen Psychosocial Questionnaire (COPSOQ) [22, 23]. Total scores for these variables range from 0 to 100 points. Workplace bullying will be assessed with a single 5-point item. The total score ranges from 0 to 100 points.

#### Other measures

Data for other variables will be collected at baseline in order to provide a description of the study sample at the end of the two-day diagnostics, to obtain the received therapy dose, and at the six-month follow-up to obtain an overall rating of interventions elements and information on healthcare utilization during the intervention and waiting period (Table 2).

##### Work Ability Index

Work ability is assessed using the German version of the WAI questionnaire [20, 21]. The total WAI score ranges from 7 to 49 points. Higher scores indicate better work ability. Levels of work ability can be categorized as poor (7–27 points), moderate (28–36 points), good (37–43 points), and excellent (44–49 points).

##### Healthcare utilization

Outpatient visits to physicians will be assessed at baseline as the number of visits in the last 12 months. Hospitalization within the last 12 months will be captured at baseline as the number of visits in the last 12 months.

Outpatient therapy (e.g., physiotherapy, psychotherapy or stress management training) will be assessed at six-month follow-up as the number of therapy units in the six months since the initial consultation. The use of in- and outpatient rehabilitation in the last six months will be assessed at six-month follow-up using an adapted item from the German Questionnaire for Health-Related Resource Use in an Elderly Population (Fragebogen zur Inanspruchnahme medizinischer und nicht-medizinischer Versorgungsleistungen im Alter, FIMA) [32].

##### Socio-demographic and work-related data 

We will ask participants for socio-demographic data (age, gender, native language, educational level, partnership and children), and work-related data (job position, job title, weekly working hours, fixed-term job contracts, temporary work, size of company and shift work).

##### Self-evaluation of functional capacity

The self-evaluation of functional capacity will be assessed using the Modified Spinal Function Sort (M-SFS) [30, 31]. The M-SFS measures self-efficacy in performing work-related demands and contains 20 drawings with simple written descriptions of the demands. Participants will rate their self-efficacy for each demand on a 5-point scale including 4 (able), 3, 2 or 1 point (restricted) or 0 points (unable). Items are summed to obtain a total score ranging from 0 to 80. Higher scores indicate greater self-efficacy to perform the tasks.

##### Delivered dose

Occupational health physicians document the intervention components (i.e., initial and follow-up consultations) in a standardized manner using computerized sheets. The study coordinators document the two-day comprehensive diagnostics (i.e. duration of diagnostic and therapeutic elements) in a standardized manner using computerized sheets.

##### Received dose

The participants will rate five items on the received content of the initial consultation, six items on the received content of the two-day diagnostics, five items on action skills, seven items on the consistency of the intervention (e.g., workplace orientation, comprehensiveness) and five items on the subjective goal achievement. The ratings of these items use a 4-point scale from 0 (do not agree) to 3 (completely agree). Finally, we will also ask the participants to rate the different components of our intervention strategy (i.e., initial consultation, two-day diagnostics and follow-up consultations), with grades from 1 (very good) to 5 (insufficient).

### Participant timeline

Table 3 shows the full schedule of enrollment, interventions and assessments.

**Table 3:** Schedule of enrollment, intervention, and assessments

*Timepoint*	*Initial consultation*	*Randomization*	*Two-day diagnostics*	*Follow-up consultations*	*Six months after initial consultation*	*Two-day diagnostics*	*Follow-up consultations*
**Enrollment**							
Screening and information letter	X						
Randomization		X					
**Interventions**							
Intervention group	X		X	X			
Waiting-list control group	X					X	X
**Assessments**							
Baseline questionnaire	X						
Questionnaire at the end of the two-day diagnostics			X			X	
Six-month follow-up questionnaire					X		
Computerized documentation by occupational health physicians	Continuously						
Participant interviews				X			X

### Sample size

A total number of cases of 128 persons, (64 persons per intervention arm) is necessary (two-sided type I error rate: 5%, power: 80%) in order to ensure a difference of 1 point on the Work Ability Score (standard deviation = 2). The standard deviation was estimated according to comparable studies [21, 33–35]. Although we will use multiple imputations to perform an intention-to-treat analysis, we will increase the sample size to compensate for the potential loss of participants during our follow-up assessments. This ensures sufficient power even if only complete cases are analyzed. Assuming a response rate of 60% after six months, we will recruit 210 patients in total: 105 patients per group.

Of the 210 persons recruited, 18 participants (nine from each of the intervention and control group) will be recruited to take part in interviews.

### Recruitment

Potential participants for the intervention will be identified in the cooperating companies by the responsible occupational health physician. Those employed in the cooperating companies for at least six months and who have health impairments and limited work ability are included. The proposal to include these employees in the study can come from various sources: the manager, the occupational health management, works council, occupational health physician or the employees themselves. Participation is voluntary for the employees. The occupational health physician is responsible for inclusion in the project.

The occupational health physician will distribute the study documents as information for the employees in the initial interview. An information letter will detail the content and objectives of the study, as well as the employee’s personal rights regarding the handling of personal data. Participants will give their informed consent and complete the baseline questionnaire. If the follow-up questionnaire is not returned, a questionnaire will be sent again, with a reminder to all participants after three weeks.

The study coordinators will carry out recruitment for the interviews in the rehabilitation centers. Informed consent forms will be handed out at the end of the two-day diagnostics. Participants will also receive a contact form that they can complete if they agree to participate in interviews. The completed contact form will be sealed by participants in a prepaid envelope addressed to the University of Lübeck. After receiving the contact form, the University of Lübeck will contact the participants and arrange an interview date.

### Allocation

A separate randomization sequence will be created by the principal investigator (MB) for each rehabilitation center using Stata 16.0. Blocks of four and six will be combined in the computer-generated randomization lists, in order to guarantee balanced case numbers, even if the lists cannot be processed completely. The randomization envelopes are consecutively numbered and non-transparently sealed.

Participants will be informed that there are two different study groups (intervention and control group), and that allocation to the two groups is randomized, during the initial consultation with the occupational health physician. After a participant has given their consent, the occupational health physician will contact the study coordinator and register them for the two-day diagnostics. The study coordinator will then open the randomization letter and communicate the group assignment to the occupational health physician and the participant by telephone, and coordinate the start of the two-day diagnostics.

### Blinding

Occupational health physicians conduct the initial consultation without knowing the group assignment. After randomized allocation no one will be blinded during or after the trial, as the realized intervention will be recognizable for all stakeholders. The principal investigator and data analysts will be aware of group assignments when analyzing the data.

### Data collection

Outcomes and other measures will be assessed with patient questionnaires based on reliable, valid, and responsive instruments (see Table 2). Baseline questionnaires and a return envelope addressed to the University of Lübeck will be handed to the participants during the initial consultation by the trained occupational health physicians. Patient questionnaires for the six-month follow-up will be sent by mail to the participants six months after random assignment by the three rehabilitation centers with a return envelope addressed to the University of Lübeck. The questionnaires at the end of the two-day diagnostics will handed out to the participants by the study coordinators in the rehabilitation centers together with a return envelope addressed to the University of Lübeck. The patient forms and questionnaires have been tested in a previous pilot study.

The occupational health physicians will be trained for the required computerized documentation before the randomized controlled trial begins. The occupational health physicians will send the computerized documentation of the initial and follow-up consultations quarterly to the researchers, who will check it for completeness and validity.

The study coordinators will document the two-day comprehensive diagnostics and send the pseudonymized computerized documentation to the researchers after completion of the diagnostics.

If participants withdraw their consent, the collected data will be deleted. A single reminder will be sent three weeks after the first mailing of the six-month follow-up questionnaires, again containing the questionnaire and the return envelope addressed to the researchers.

We have created a website to inform study participants about the study, and to maintain interest in the study.

Interviews will take place after the two-day diagnostics, during the process of the follow-up consultations.

### Data management

A comprehensive data protection concept has been developed with the data protection officer from German Pension Insurance North which clarifies the data processing, the rights of participants, and technical and organizational measures in order to ensure the secure and confidential collection, processing, and storage of data. Data from the questionnaires will be entered, reviewed and exported to statistical software packages for further analysis. Data input and data verification will be performed by trained research assistants.

Recordings from the interviews will be transcribed by trained assistants at the University of Lübeck. Names and places will be removed during the transcription process.

Access to the data is limited to the first and last author and research assistants on the research team, and data management is performed by these authors.

### Statistical methods

Linear mixed models will be used for continuous outcomes and logistic mixed models for binary outcomes. We will include a random intercept to consider varying outcomes in different rehabilitation centers.

Baseline scores of outcomes will be included as covariates. In order to perform an intention-to-treat analysis, we will use multiple imputation to augment incomplete responses to the six-month follow-up questionnaires. Exploratory moderator analyses examine whether estimates differ for sex, job position, size of company, and the rehabilitation center [36].

We will not perform interim analyses or specify a stopping rule. Statistical tests will be regarded as significant if the two-sided *p*-value is less than 0.05. An up-to-date version of Stata (StataCorp, College Station, Texas, USA) will be used to conduct analyses.

The interviews will be transcribed and analyzed using qualitative content analysis.

## Discussion

The purpose of our randomized controlled trial is to test the effects of a new complex intervention that contains a comprehensive diagnostic service with medical, physiotherapeutic and psychotherapeutic elements to clarify the need for interventions for workers with health problems whose work participation is at risk with tailored interventions early enough to prevent the worsening of health and work ability. Updated information is provided on our trial website: www.gibi-rehapro.de. The results of our study will be published as articles in peer-reviewed journals and at conferences. The authors of this protocol will write the final trial publications. We do not intend to use professional writers. The researchers and German Pension Insurance North will design a flyer providing information about the key findings of our study (circulation: 2000 copies). These will be distributed nationwide in Germany. We will also host a symposium to provide information about our study.

The study protocol was designed using the SPIRIT (Standard Protocol Items: Recommendations for Interventional Trials) checklist [37].

### Trial status

Recruitment has started and is ongoing.

## Supplementary Information

**Additional file 1.** Items from the World Health Organization Trial Registration Data Set.

**Additional file 2.** Information on participation in the randomized controlled trial.

**Additional file 3.** Consent form for the randomized controlled trial.

**Additional file 4.** Information on the six-month follow-up for the intervention group of the randomized controlled trial.

**Additional file 5.** Information on the six-month follow-up for the control group of the randomized controlled trial.

## Data Availability

We will share all individual participant data that underlies the results of our primary publication. This data will be completely anonymized. We will provide a link to our study registration. Data will be available immediately and indefinitely after our primary publication is accepted. Anyone who wishes can access the data from https://www.synapse.org/.

## Abbreviations

BSA-F: Bewegungs- und Sportaktivität Fragebogen
COPSOQ: Copenhagen Psychosocial Questionnaire
FEBA: Fragebogen zur subjektiven Einschätzung der Belastungen am Arbeitsplatz
FIMA: Fragebogen zur Inanspruchnahme medizinischer und nicht-medizinischer Versorgungsleistungen im Alter
GIBI: Comprehensive clarification of the need for intervention in persons whose work participation is at risk
ICF: International Classification of Functioning, Disability and Health
M-SFS: Modified Spinal Function Sort
PHQ: Patient Health Questionnaire
RMQ: Roland and Morris Disability Questionnaire
SPIRIT: Standard Protocol Items: Recommendations for Interventional Trials
TIDieR: Template for Intervention Description and Replication
WAI: Work Ability Index
WAS: Work Ability Score
