# Supplementary material for: A comprehensive diagnostic service to clarify intervention needs when work participation is at risk: study protocol of a randomized controlled trial (GIBI, DRKS00027577)
Source: BMC Health Serv Res. 2022 Sep 9;22:1142. doi: 10.1186/s12913-022-08513-1 (PMC9463831; doi:10.1186/s12913-022-08513-1)
Supplement: Supplementary file 1 — Additional file 1. Items from the World Health Organization Trial Registration Data Set. [file 12913_2022_8513_MOESM1_ESM.docx]

# Additional file 1: Items from the World Health Organization Trial Registration Data Set

| **Data category** | Information |
| --- | --- |
| Register | German Clinical Trials Register |
| Last refreshed on | 2022/02/01 |
| Main ID | DRKS00027577 |
| Date of registration | 2022/02/01 |
| Prospective Registration | Yes |
| Primary sponsor | Universität zu Lübeck, Institut für Sozialmedizin und Epidemiologie |
| Public title | Comprehensive clarification of the need for intervention for people whose work participation is at risk |
| Scientific title | Comprehensive clarification of the need for intervention for people whose work participation is at risk – GIBI |
| Date of first enrolment | 2022/05/30 |
| Target sample size | 210 |
| Recruitment status | Pending |
| URL | https://www.drks.de/drks_web/navigate.do?navigationId=trial.HTML&TRIAL_ID=DRKS00027577 |
| Study type | Interventional |
| Allocation | Randomized controlled trial |
| Masking | Open (masking not used). |
| Control | Control group receives no treatment. |
| Assignment | Parallel |
| Purpose | Treatment |
| Phase | N/A |
| Countries of recruitment | Germany |
| Contacts | David Fauser Ratzeburger Allee 160, 23562 Lübeck, Germany +49 451 929951 19 [davidpeter.fauser@uksh.de](mailto:davidpeter.fauser@uksh.de)  Universität zu Lübeck, Institut für Sozialmedizin und Epidemiologie |
| Key inclusion and exclusion criteria | Inclusion criteria: Included are employees with health restrictions and limited ability to work who have been on sick leave for at least 4 weeks in the past 12 months, have been employed in the cooperating companies for at least 6 months and are insured with the German Pension Insurance North, Federal German Pension Insurance, German Pension Insurance Braunschweig-Hannover or German Pension Insurance Knappschaft-Bahn-See.  Exclusion criteria: Excluded are individuals who require urgent medical care due to acute illnesses, who have a clear need for rehabilitation services or who initially need support due to an addiction.  Age minimum: 18 Years  Age maximum: 65 Years  Gender: Both, male and female |
| Health conditions or problems studied | Chronic health problems |

| Interventions | Intervention group:  Identification of potential participants: Potential participants for the intervention are identified in the cooperating companies by the responsible occupational health physician. The target group are employees who have been employed in the cooperating companies for at least six months and begin to have health impairments that lead to subjective (fears of the occupational health physician) or objective indications (e.g., frequent periods of sickness absence) that individual capacity and professional requirements are increasingly drifting apart although the exact reason for this remains unclear. The initiative to include these employees in the project can come from different places: from the manager, from a steering committee responsible for occupational health management, from the works council, from the occupational health service or from the employees themselves.  Initial consultation with the occupational health physician: The occupational health physician arranges an initial consultation with potential participants and interested employees. During this consultation, the occupational health physician clarifies the suitability of the employees and describes the planned procedure. The employees are informed about the scientific study and asked to participate.  Two-day long participation assessment: The two-day comprehensive diagnostics are carried out by a multi-professional rehabilitation team. The results of the physiotherapeutic and psychosomatic diagnostics are integrated into a standardized profile comparison between job demands and individual capacity using the documentation procedure IMBA (IMBA is a German acronym for „Integration of People with Disabilities into the Working Environment”). Recommendations for possible further measures are derived from the profile comparison, communicated to the participants, and forwarded to the responsible occupational health physician. In addition to diagnostic elements, the intervention also contains sections aimed at activation and empowerment. During the stay, it is possible to participate in offers of the rehabilitation centers with the focus on strengthening self-care competences.  Follow-up consultations with the occupational health physician: The employee and the occupational health physician exchange information about the results of the intervention, further measures, and additional support needs. The occupational health physician has an important role of accompaniment, stabilization, and motivation of the employee. Depending on the needs, up to four meetings with the occupational health physician should be scheduled within six months of the two-day stay in the rehabilitation center. If necessary, the occupational health physician can clarify open questions with the involved health professionals in the rehabilitation center.  Control group:  The wait list control group is identified in the same way as the intervention group and will receive the initial consultation with the occupational health physician. The control group will receive the two-day comprehensive diagnostics at the rehabilitation center not before six months after the initial consultation with the occupational health physician. |
| --- | --- |
| Primary outcome | The primary outcome measure is the Work Ability Score. The Work Ability Score is the first item of the Work Ability Index and measures the current self-assessed work ability compared to the best work ability ever achieved (0-10 points). This variable is recorded in the initial interview and six months after the initial interview. |
| Secondary outcomes | Various secondary outcomes are collected to reflect changes in health and participation: general health (0 to 10 points, Nuebling et al. 2010), depression (0 to 6 points, Low et al. 2010), anxiety (0 to 6 points, Lowe et al. 2010), physical functioning (0 to 24 points, Roland et al. 2000), self-rated work ability (7 to 49 points, Ilmarinen 2007), days of sickness absence in the past six months, current employment, physical job demands (0 to 15 points, Slesina, 1987), mental job demands (0 to 100 points, Nuebling et al. 2010), social support at work (0 to 100 points, Nuebling et al. 2010), work atmosphere (0 to 100 points, Nuebling et al. 2010), job insecurity (0 to 100 points, Nuebling et al. 2010), bullying (0 to 100 points, Nuebling et al. 2010), job satisfaction (0 to 100 points, Nuebling et al. 2010). These data are collected in the initial consultation and six months after the initial interview.  Six months after the initial consultation, the intervention group, which began the two-day diagnostics promptly after being included into the study, is also surveyed to determine what content the participants have received and how they evaluate the elements of the intervention (with regards to their own development): consistency of intervention (0 to 21 points), subjective achievement of goals (0 to 15 points), evaluation of initial consultation (1 to 6 points), evaluation of two-day diagnostics (1 to 6 points), evaluation of the follow-up care consultations (1 to 6 points) and evaluation of the overall offer (1 to 6 points).  At the end of the two-day diagnostics, the self-evaluation of functional capacity (SELF, 0 to 80, Janssen et al. 2016), perceived content of the initial consultation (0 to 15 points), perceived content of the two-day diagnostic measure (0 to 18 points) and capacity to act (0 to 20 points) are collected.  The occupational health physicians and specialists in the rehabilitation centers additionally document which diagnostic and therapeutic services were provided in the rehabilitation centers (quantity and duration in minutes). |
| Secondary ID(s) | 21-503 (Ethics Committee University of Lübeck)  U1111-1271-2163 (Universal Trial Number) |
| Source of monetary support | Knappschaft-Bahn-See - Fachstelle rehapro im Auftrag des Bundesministeriums für Arbeit und Soziales |
| Status of ethics review | Approved |
| Approval date of ethics review | 14/02/2022 |
