# Supplementary material for: A comprehensive diagnostic service to clarify intervention needs when work participation is at risk: study protocol of a randomized controlled trial (GIBI, DRKS00027577)
Source: BMC Health Serv Res. 2022 Sep 9;22:1142. doi: 10.1186/s12913-022-08513-1 (PMC9463831; doi:10.1186/s12913-022-08513-1)
Supplement: Supplementary file 2 — Additional file 2. Information on participation in the randomized controlled trial. [file 12913_2022_8513_MOESM2_ESM.docx]

# Information

**to participate in the scientific study accompanying**

**GIBI - Comprehensive clarification of the need for intervention in persons whose work participation is at risk**

## What do we offer you?

The causes of health problems are often complex, and sometimes different health problems interact and reinforce each other. To understand the effects of health problems, we must also look at the individual life and work situation. The GIBI study provides you with a professional and comprehensive diagnosis. Depending on your place of residence, the Fachklinik Aukrug (Tönsheide 10, 24613 Aukrug), the RehaCentrum Hamburg (Heidenkampsweg 41, 20097 Hamburg) and the Zentrum für ambulante Rehabilitation in Rostock (Wismarsche Str. 32, 18057 Rostock) are available to you for the two-day diagnostics. The rehabilitation centers support you in developing solutions that are suitable for you. The three-stage measure is initiated by your occupational health physician:

1. The occupational health physician assesses your health restrictions in an initial consultation and describes your specific job demands.
2. You will then receive a comprehensive two-day diagnostic test at the rehabilitation center in order to derive action strategies to secure your employment and improve your work ability.
3. Your occupational health physician will monitor the implementation of the action strategies in up to four follow-up consultations with you. The consultations are held within the first six months after the comprehensive two-day diagnostics.

## What questions does the scientific study clarify?

The aim of the scientific study is to clarify the effectiveness of the intervention. Of course, we do not want to deprive you of our intervention, however, in order to be able to reliably assess the effectiveness of the intervention, one group will start the intervention promptly and another group will start the intervention six months later. We can then compare these two groups. Assignment to one of these two groups must be made by chance. If you decide to participate in the study, there is a 50% chance in each case that you will receive the intervention either immediately or six months later. The decision will be made randomly. The University of Lübeck has prepared envelopes for this purpose, which only inform participants about their assignment to one of the two groups when they are opened. Whatever the assignment, you will be informed of the decision in a timely manner. The ethics committee of the University of Lübeck has reviewed the plan to conduct the scientific study.

## General information on data protection

In accordance with the European Data Protection Regulation (EU-DSGVO), we would like to inform you about the data we collect during the intervention and the purpose of the data we collect. We will also inform you about your rights.

## What data is collected?

The participating occupational health physicians and the professionals in the rehabilitation center will conduct written surveys with you, which will be evaluated by the University of Lübeck. For the first survey, you will be given a questionnaire by your occupational health physician. You will receive another questionnaire by mail six months after the start of the program, regardless of the group assignment described above. The questionnaires focus on your health and work ability and will be sent by you directly to the University of Lübeck, in a postage-paid envelope). During your stay in one of the three rehabilitation centers, a written survey will also be conducted in which you can evaluate the services offered. The University of Lübeck will be sent this survey by the professionals at the rehabilitation center.

Those involved in the project will also document the new intervention (e.g., time, duration and type of diagnostic measures). We would also like to interview some participants personally during the study.

## How do we handle the data we collect?

Your occupational health physician will handle the results of the study in confidence, and will also have a duty of confidentiality towards your employer in our study. A study number will be assigned to your name to protect your data. All evaluations are therefore pseudonymized: they are carried out with knowledge of your identification number only, and without your name or other personal details. To ensure a transparent research process, the pseudonymized data are permanently stored in a data repository (data archive) (https://www.synapse.org/). All data at the University of Lübeck will be completely deleted after ten years.

## Voluntary participation and your rights

Participation in the model project and the scientific study is voluntary. If you wish to withdraw your participation at a later date, obtain information about your stored data, assert your right to restrict the processing of your data or exercise your right to data deletion, please contact David Fauser at the University of Lübeck

(E-mail: davidpeter.fauser@uksh.de). Withdrawal will result in the permanent deletion of the collected data. The deletion of data after withdrawal is only possible until 30th June 2024, the date of the data’s complete anonymization. Participation in the intervention without participation in the scientific study is not possible. If study participation is withdrawn, the intervention will therefore also be terminated prematurely.

In the event of a complaint, please contact:

Unabhängiges Landeszentrum für Datenschutz Schleswig-Holstein

Holstenstraße 98, 24103 Kiel, E-Mail: mail@datenschutzzentrum.de

## Responsible body for the research project and responsible data protection officer

Responsibility for the research project lies with Prof. Dr. Matthias Bethge of the University of Lübeck (E-mail: matthias.bethge@uksh.de). If you have any questions about data protection, please contact the data protection officer at the University of Lübeck Study Center:

x-tention, Informationstechnologie GmbH, Karl-Drais-Str. 4e, 86167 Augsburg, Germany, Tel.: +49 451 3101 1903, E-mail: datenschutz@uni-luebeck.de

If you have any questions, please feel free to contact us.

We thank you for your participation and send you our kind regards.


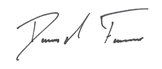


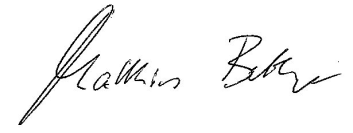


Matthias Bethge David Fauser
