# Supplementary material for: A comprehensive diagnostic service to clarify intervention needs when work participation is at risk: study protocol of a randomized controlled trial (GIBI, DRKS00027577)
Source: BMC Health Serv Res. 2022 Sep 9;22:1142. doi: 10.1186/s12913-022-08513-1 (PMC9463831; doi:10.1186/s12913-022-08513-1)
Supplement: Supplementary file 3 — Additional file 3. Consent form for the randomized controlled trial. [file 12913_2022_8513_MOESM3_ESM.docx]

# Consent

**to participate in the scientific study accompanying**

**GIBI -** **Comprehensive clarification of the need for intervention in persons whose work participation is at risk**

Surname Name

I have been informed about the content and the aim of the GIBI study. The GIBI study is funded by the Federal Ministry of Labour and Social Affairs as part of the Rehapro federal funding program (<https://www.modellvorhaben-rehapro.de>).

The accompanying study is headed by Prof. Dr. Mathias Bethge from the University of Lübeck, who will conduct the study and evaluate the data. A leaflet with information about data protection was given to me.

I would like to support the study through my participation and agree to complete the questionnaires that will be given to me. There will be no cost to me.

If I am willing to participate in an interview, I can send my contact details to the University of Lübeck and arrange an interview date. Participation in the study is also possible without taking part in the interviews.

An exchange of data between the occupational health physician and the rehabilitation center is necessary for the implementation of the measure. I consent to the occupational health physician transmitting the medical information required to carry out the measure to the rehabilitation center in compliance with data protection regulations. I also consent to the rehabilitation center transmitting the results of the measure to the occupational health physician in the form of a final report in compliance with data protection regulations. The parties involved will act in accordance with their existing data protection regulations.

I agree that the data from the documentation of the study coordinators in the rehabilitation center (e.g., time, duration, and type of diagnostic measures) and my questionnaire data will be merged by the researchers using an identification number.

I have been assured that no personal data (name, year of birth, contact details) or other information allowing conclusions to be drawn about my person will be passed on to third parties outside the study.

I understand that I can withdraw my consent at any time without giving reasons and without disadvantage. I have been fully informed about my rights, and also about the time I will spend as a study participant. All my questions have been answered to my satisfaction.

Under these conditions, I give my consent to participate in the study.

Place, data Signatur
