# Supplementary material for: A comprehensive diagnostic service to clarify intervention needs when work participation is at risk: study protocol of a randomized controlled trial (GIBI, DRKS00027577)
Source: BMC Health Serv Res. 2022 Sep 9;22:1142. doi: 10.1186/s12913-022-08513-1 (PMC9463831; doi:10.1186/s12913-022-08513-1)
Supplement: Supplementary file 4 — Additional file 4. Information on the six-month follow-up for the intervention group of the randomized controlled trial. [file 12913_2022_8513_MOESM4_ESM.docx]

Name

Address

Lübeck, yyyy-mm-dd

Final survey on the GIBI scientific study

Dear Ms. ..., Dear Mr. ...,

About six months ago, you received an invitation from your occupational health physician to participate in the new GIBI intervention. Let me remind you once again: GIBI is a model project that the University of Lübeck has developed together with German Pension Insurance North, the Fachklinik Aukrug, the RehaCentrum Hamburg and the Zentrum für ambulante Rehabilitation in Rostock in order to improve the ability of people with health restrictions to work. For this purpose, you were given a two-day stay at one of our participating rehabilitation centers, where individually suitable action measures were created for you.

**Why do we write to you today?**

We need your support in order to evaluate the effectiveness of the GIBI intervention. We would therefore like to ask you to complete the enclosed questionnaire.

**What can you do?**

Please complete the enclosed questionnaire independently and completely, and then put it into the enclosed return envelope (postage paid by recipient). Send the envelope directly to the University of Lübeck.

**Voluntary participation**

We would like to remind you once again at this point that your participation in the survey is voluntary. If you decide not to participate, you do not need to do anything. You will not suffer any disadvantage as a result. You can also simply ignore the reminder letter that will reach you in about three weeks.

**Request for participation**

We will be very pleased if you participate in the survey. Your information is very valuable to us in order to improve and further develop our services.

If you have any questions, please feel free to contact us.

We thank you for your participation and send you our kind regards.

Rehabilitation center
